# Expression of Rift Valley fever virus N-protein in *Nicotiana benthamiana* for use as a diagnostic antigen

**DOI:** 10.1186/s12896-018-0489-z

**Published:** 2018-12-11

**Authors:** Sandiswa Mbewana, Ann E. Meyers, Brandon Weber, Vuyokazi Mareledwane, Maryke L. Ferreira, Phelix A. O. Majiwa, Edward P. Rybicki

**Affiliations:** 1 Biopharming Research Unit, Department of Molecular and Cell Biology, University of Cape Town, 6503200115084, Rondebosch, Cape Town, 7700 South Africa; 2 Structural Biology Research Unit, University of Cape Town, P Bag X3, Rondebosch, 7700 South Africa; 3 ARC-Onderstepoort Veterinary Institute, 100 Old Southpan Road, Onderstepoort, 0110 South Africa; 4 Institute of Infectious Disease and Molecular Medicine, University of Cape Town, Anzio Road, Observatory, Cape Town, 7925 South Africa

**Keywords:** Rift Valley fever virus, N-protein, Diagnostic, ELISA, DIVA, Molecular farming, Plant production

## Abstract

**Background:**

Rift Valley fever virus (RVFV), the causative agent of Rift Valley fever, is an enveloped single-stranded negative-sense RNA virus in the genus *Phlebovirus*, family *Bunyaviridae*. The virus is spread by infected mosquitoes and affects ruminants and humans, causing abortion storms in pregnant ruminants, high neonatal mortality in animals, and morbidity and occasional fatalities in humans. The disease is endemic in parts of Africa and the Arabian Peninsula, but is described as emerging due to the wide range of mosquitoes that could spread the disease into non-endemic regions.

There are different tests for determining whether animals are infected with or have been exposed to RVFV. The most common serological test is antibody ELISA, which detects host immunoglobulins M or G produced specifically in response to infection with RVFV. The presence of antibodies to RVFV nucleocapsid protein (N-protein) is among the best indicators of RVFV exposure in animals. This work describes an investigation of the feasibility of producing a recombinant N-protein in *Nicotiana benthamiana* and using it in an ELISA.

**Results:**

The human-codon optimised RVFV N-protein was successfully expressed in *N. benthamiana* via *Agrobacterium*-mediated infiltration of leaves. The recombinant protein was detected as monomers and dimers with maximum protein yields calculated to be 500–558 mg/kg of fresh plant leaves. The identity of the protein was confirmed by liquid chromatography-mass spectrometry (LC-MS) resulting in 87.35% coverage, with 264 unique peptides. Transmission electron microscopy revealed that the protein forms ring structures of ~ 10 nm in diameter. Preliminary data revealed that the protein could successfully differentiate between sera of RVFV-infected sheep and from sera of those not infected with the virus.

**Conclusions:**

To the best of our knowledge this is the first study demonstrating the successful production of RVFV N-protein as a diagnostic reagent by *Agrobacterium*-mediated transient heterologous expression in *N. benthamiana*. Preliminary testing of the antigen showed its ability to distinguish RVFV-positive animal sera from RVFV negative animal sera when used in an enzyme linked immunosorbent assay (ELISA). The cost-effective, scalable and simple production method has great potential for use in developing countries where rapid diagnosis of RVFV is necessary.

**Electronic supplementary material:**

The online version of this article (10.1186/s12896-018-0489-z) contains supplementary material, which is available to authorized users.

## Background

Rift Valley fever (RVF) is a mosquito-borne viral zoonosis caused by Rift Valley fever virus (RVFV). This is an enveloped virus belonging to the genus *Phlebovirus* of the family *Bunyaviridae*. It has a three-component single-stranded RNA genome comprising of a large segment (L), a medium segment (M), and a small (S) segment, each enclosed in a separate helical nucleocapsid (N-protein) within the virion (Garcia et al., 2001).

The requirement for countries to monitor and manage RVFV outbreaks within their borders has become ever more urgent due to global warming, which has expanded the regions habitable by the mosquito vector. Thus, there is an increased need for diagnostic assays which are reliable, accurate and rapid, for control and management of spread of RVF outbreaks. Preferred assays are those which can differentiate infected animals from those which have been vaccinated (DIVA), and those which can distinguish infected animals from uninfected ones [1]. For a zoonotic disease, such assays should work for both humans and animals.

There are several methods of detecting RVFV infections. Quantitative real-time PCR of RVFV RNA can be used to detect viraemic animals [2–5]. Additionally, a real-time reverse transcription loop-mediated isothermal amplification (LAMP) test for the rapid detection of RVFV has been developed [6–8]. More recently, a method using single step real-time reverse transcription-polymerase chain reaction for the detection of RVFV RNA has been developed [9]. Although viraemia in individuals infected with RVFV reaches high levels, it is short-lived. This makes monitoring the spread of the virus in the field using PCR unfeasible as it could under-estimate the actual incidence. In addition, PCR requires specialised laboratory equipment and well-trained personnel, which are additional drawbacks when an outbreak occurs, especially in remote areas.

Serological tests provide a plausible alternative to PCR-based methods. Traditional methods for detection of antibodies to RVFV are haemagglutination inhibition, immunofluorescence assays and virus neutralization (VN), which is regarded as the gold standard [10]. Disadvantages of these tests include the high health risk to laboratory workers who have to handle the live virus required for these tests, and the fact that the use of these tests is restricted outside RVF endemic areas because of the possibility of spreading the live virus [11, 12]. These have led to the development of highly sensitive and specific indirect enzyme-linked immunosorbent assays (ELISAs) for the detection of IgG and IgM antibodies to RVFV in the sera of animals and humans suspected of exposure to the virus. The first generation ELISAs for RVF involved the use of purified, inactivated sucrose-acetone protein extracted from tissue-cultured RVFV as the coating antigen [13–15]. However, the use of such inactivated antigens has several disadvantages: they tend to bind poorly to ELISA plates [16], and production of the antigen also requires the use of a high level biosafety containment facility, which increases the cost of production and the risk of exposure to laboratory personnel [16, 17]. Furthermore, these ELISAs cannot be used in areas free of RVF because the antigen is derived directly from live virus and the risk of viral escape into naïve areas. Therefore, there is a continuing need for a cheap, safe and more consistent test for screening animal and human sera for possible presence of RVFV antibodies.

RVFV N-protein is highly conserved amongst RVFV strains [18–20], and is the main immunodominant viral protein in other members of the *Bunyaviridae* family (Schwarz et al., 1996; Swanepoel et al., 1986). RVFV N-protein has been successfully produced in *Aedes pseudoscaullaris* mosquito cell lines [20]; *Trichoplusia ni* (Tn5) insect cells [21] and in *E. coli* [16, 17, 22, 23]. At least two of the commercially available RVFV competition ELISAs - BDSL (Ayrshire, UK) and ID-VET (Montpellier, France) - incorporate recombinant *E. coli*-produced N-protein for the detection of RVFV antibodies in animal serum [24]. A double antigen ELISA for the simultaneous identification of IgM and IgG of RVFV antibodies, using N-protein produced in *E. coli,* has also been developed and validated using sheep and cattle serum samples [25]. There is currently no universally accepted detection assay. The commercially available multi-species IgG and IgM ELISAs are very expensive, and thus not suitable for routine use [26].

Despite the success of *E. coli*-produced N-protein in ELISA, there are some disadvantages: *E. coli*-made recombinant proteins are often incorrectly folded and/or often insoluble or aggregated, which may influence their utility in the ELISA and subsequent sensitivity [27]. It is therefore more desirable to use N-protein which is properly folded to facilitate the maximum possible binding of antibodies. Recombinant proteins produced in plants are usually correctly folded and post-translationally modified because it is a eukaryotic expression host [28]. In addition, a plant production system is free of contaminating animal-derived agents, which is often a major concern; it is safe to handle during production, more cost effective than other expression platforms and allows for rapid, scalable production of recombinant proteins ([28–32], Merlin, 2014 #225). RVFV N-protein has recently been expressed in transgenic *Arabidopsis thaliana* [33]: however, this was intended for use as a vaccine candidate and yields were relatively low, ranging from levels of 3.3 μg/g in root tissue to 3.8 μg/g fresh weight in leaves.

This is the first report of the high-yielding production of RVFV N-protein in *Nicotiana benthamiana*, and its evaluation in a validated ELISA using sheep sera of known RVF status. This recombinant protein when used in ELISA can differentiate between sera from infected sheep and those from uninfected ones. The use of this recombinant protein in testing animals to determine whether or not they have been exposed to RVFV makes a significant contribution to the One Health approach to control and management of this important re-emerging zoonotic disease [34–36].

## Results

### Cloning of Rift Valley fever virus nucleocapsid (N) protein gene

**Fig. 1:**
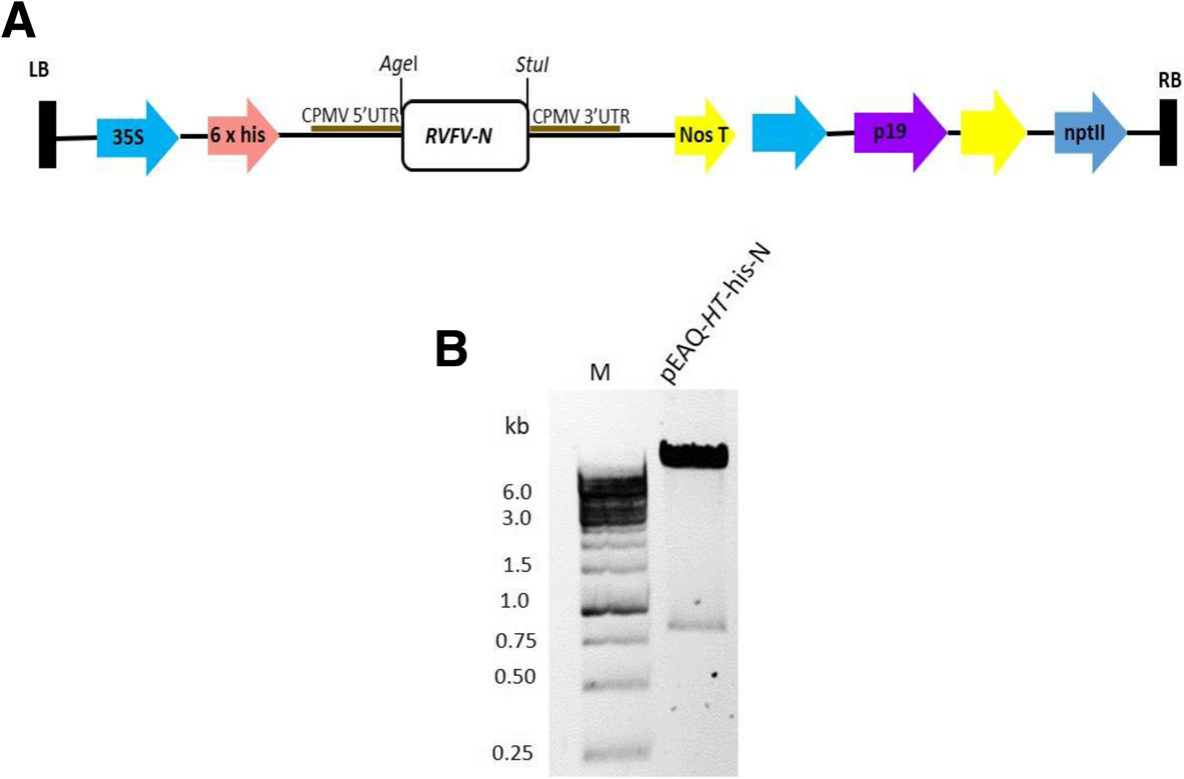
Schematic presentation and confirmation of pEAQ-*HT*-his-N. **a** pEAQ-*HT* vector components; RB and LB: right and left borders for T-DNA integration, 35S promoter from Cauliflower mosaic virus (CaMV), 5’UTR: modified 5’ UTR from CPMV RNA-2, 3’UTR from CPMV RNA-2, NosT: nopaline synthase terminator, P19: suppressor of gene silencing from TBSV, 35S terminator from CaMV, *npt*II: kanamycin resistance gene, OriV: pRK2 origin of replication, TrfA: replication essential locus and ColEI; the pBR322 *E. coli* origin of replication [48]. **b** Confirmation of pEAQ-*HT*-his-N (9947 and 773 bp) by restriction enzyme digest mapping with *Nru*I and *Stu*I, 1 kb O’GreenGene ruler (M) was used as a DNA ladder. DNA was resolved on 0.8% (*w*/*v*) TBE agarose gel

The sequence encoding the nucleocapsid of the South African RVFV isolate M35/74 (GenBank accession number JF784 388) is 750 nucleotides long with an open reading frame of 245 aa. This was cloned between the 5′ and 3′ cowpea mosaic virus (CPMV) untranslated regions (UTRs) in the plant expression vector pEAQ-*HT*, resulting in the plasmid pEAQ-*HT*-his-N (N terminal 6xHis-tag) (Fig. 1a). Correct insertion of the sequence in the recombinant plasmid was confirmed by restriction digest mapping giving the expected banding pattern of DNA fragments sized 9947 and 773 bp (Fig. 1b).

### Transient expression and detection of recombinant N-protein in crude leaf extracts

The recombinant plasmid pEAQ-*HT*-his-N was electroporated into *A. tumefaciens* LBA4404 cells. Recombinant protein expression of N-protein in leaves infiltrated with recombinant culture optical densities (OD_600_) of 0.25, 0.5 and 1 was tested. Leaves were harvested at 1–4 days post infiltration (dpi) and crude leaf extracts then subjected to western blot analyses using anti-N antibody; equal amounts of total soluble protein (30 μg) was loaded in each well.

**Fig. 2:**
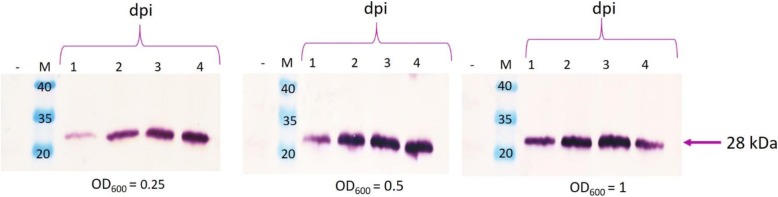
Transient expression time trial of N-protein in *N. benthamiana* by *Agrobacterium*-mediated transfer. Infiltration at OD_600_ of 0.25, 0.5, and 1. Lanes 1–4 denote the number of days post infiltration (dpi). Lane “-“, − negative control represented by extract from leaves infiltrated with pEAQ-*HT* vector lacking any gene of interest. Lane M contains PageRuler™ Prestained protein ladder (Thermo Scientific, MA, USA). The protein was detected with 1:5000 anti-N primary antibody and 1:5000 anti-rabbit secondary antibody

The expected band size of 28 kDa representing N-protein, as well as a dimer of 56 kDa, was detected in leaf samples infiltrated with pEAQ-*HT*-his-N from 1 dpi onwards (Fig. 2). At OD_600_ = 0.25 and 0.5, protein band intensity progressively increased in concentration with the incubation period until 4 dpi. At OD_600_ = 1, the protein band intensity progressively increased from 1 to 3 dpi and decreased at day 4. These protein bands were absent in the negative control lane. Qualitatively, the highest protein expression levels, as judged by the intensity of the protein bands, were seen when an *Agrobacterium* culture OD_600_ of 0.5 was used to infiltrate leaves and they were harvested at 3 and 4 dpi. The OD_600_ of 0.5 was selected for further experimentation. Since speed would be an important factor when producing this antigen commercially, the shorter 3 dpi period of incubation was preferred for further analysis of the N-protein.

### Purification of recombinant protein

**Fig. 3:**
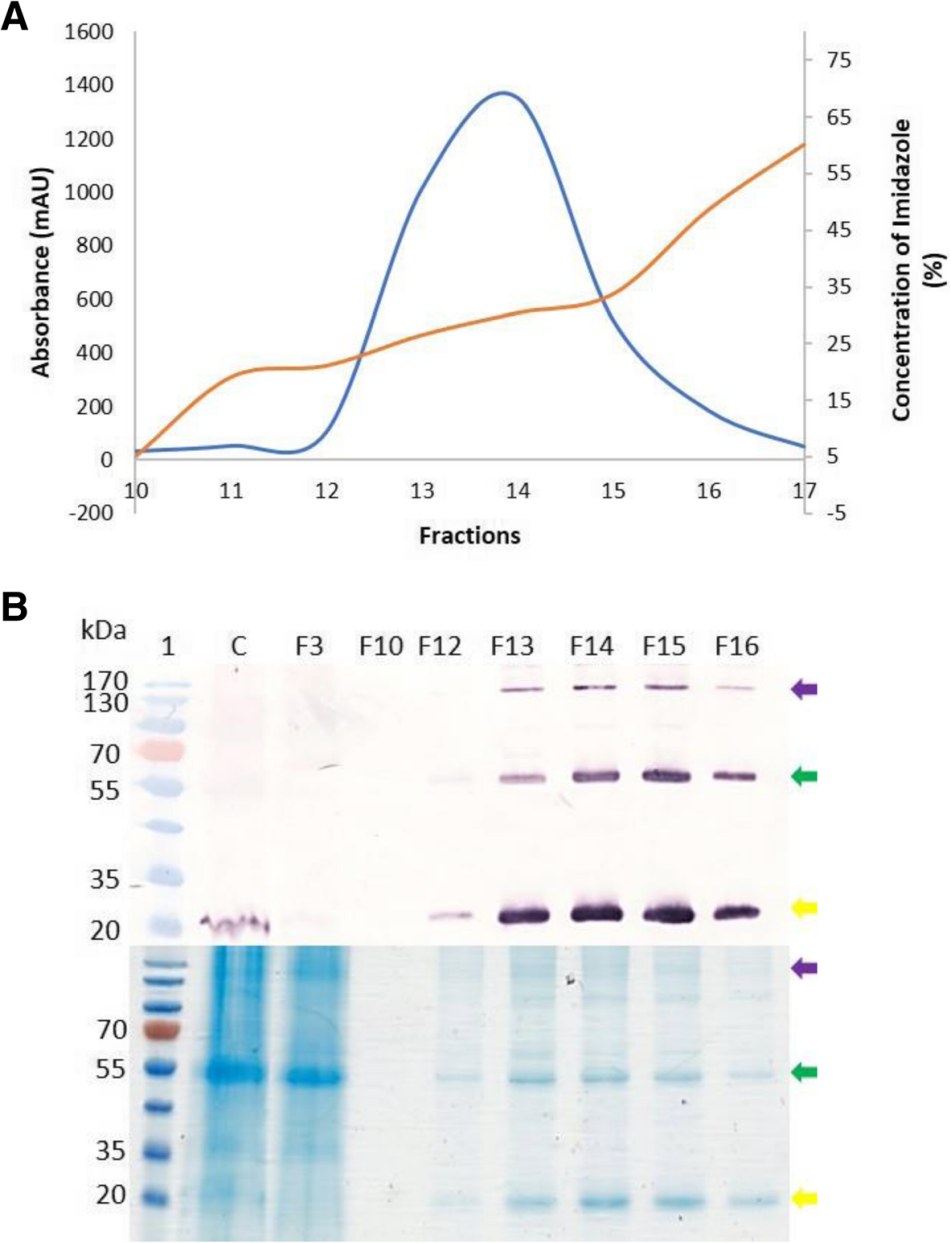
Purification of the N-protein using nickel affinity chromatography. **a** Chromatographic trace showing 6xHis-tag N-protein elution (orange line) from the 6xHis-tag-chelating affinity column with increasing imidazole concentration (blue line). **b** Western blot analysis (top) and Coomassie-stained gel (bottom) of collected fractions. Lane 1 contained PageRuler ™ Prestained protein ladder (Thermo Scientific, MA, USA), C contains crude plant extract, F3 represents unbound wash fraction 3, F10 contained a wash fraction 10, F12–16 protein peak visualised in fractions 12–16. The protein was detected with 1:5000 anti-N primary antibody and 1:5000 anti-rabbit secondary antibody. The purple arrow indicates the pentamer, the green arrow indicates the dimer and the yellow arrow indicates the monomer

To scale up the production of N-protein, 40 plants were vacuum-infiltrated at an OD_600_ of 0.5 and leaves harvested at 3 dpi. N-protein was enriched for by (NH_4_)_2_SO_4_ precipitation and subsequent nickel affinity column chromatography. The protein was detected in the 0–40% (NH_4_)_2_SO_4_ fraction and was purified by 6xHis-tag affinity chromatography. The protein was eluted from the column with an increasing gradient of imidazole concentration, corresponding to a protein peak visualised in fractions 12–16 (Fig. 3a). The recombinant protein was detected by western blot analysis as monomers (28 kDa), dimers (56 kDa) and putative pentamers (140 kDa) (Fig. 3 b). The maximum protein yield of RVFV N-protein was calculated to be ~ 500–558 mg/kg of fresh weight leaf material. The identity of the 28 kDa protein monomer was confirmed by LC-MS, resulting in 87.35% identity and 264 unique peptides (Additional file 1: Figure S1).

### Transmission Electron microscopy (TEM)

**Fig. 4:**
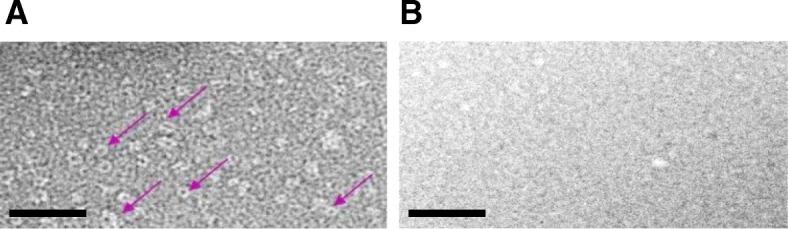
Recombinant N-protein visualization by transmission electron microscopy. **a** TEM of plant-produced N-protein – ring-shaped structures of N-protein indicated by pink arrows. **b** Negative control: TEM of sample from plant leaves infiltrated with pEAQ-*HT* lacking any gene of interest and prepared using the same method as for B. Scale bars represent 100 nm

Previously, purified N-protein expressed in a bacterial system was shown using TEM to form distinct ring-shaped particulate structures approximately 10 nm in diameter [37]. Samples of affinity–purified plant-produced N-protein were therefore subjected to TEM and shown to contain similar-shaped particles of similar magnitude (Fig. 4 a). This particulate matter was not observed in the negative control (Fig. 4 b) at the same level of magnification. The ring-shaped structures were not distinguishable as pentamers, hexamers, heptamers or octamers, as this was beyond the scope of the resolution of the microscope used.

### IgG capture ELISA

**Fig. 5:**
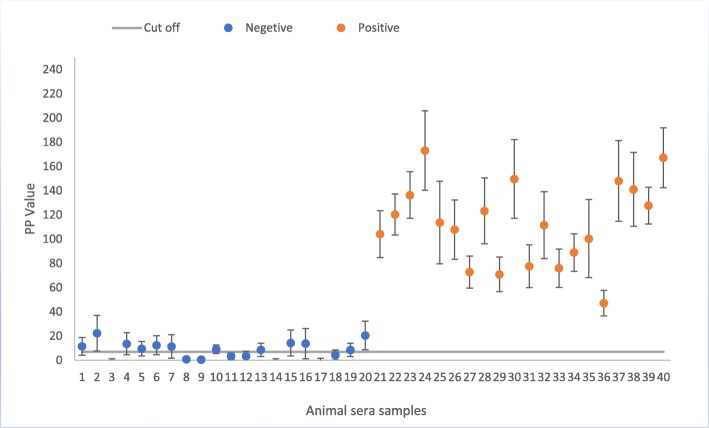
Detection of anti-RVFV IgG antibodies in sheep sera by ELISA using plant-produced N-protein. Animal serum samples 1–20 represent PP values calculated for RVFV-negative sera (blue dots) and samples 21–40 represent PP values calculated for RVFV-positive sera (orange dots). The error bars illustrate ± mean standard deviation calculated from each PP value. The cut-off value (grey line) to distinguish between positive and negative test outcome in IgG capture ELISA was calculated as the percentage of high-positive control serum (PP) values greater than 7.0. PP values less than 4.0 are regarded as negative and PP values between 4.0 and 7.0 are regarded as suspect

To determine whether the purified plant-produced RVFV N antigen can detect IgG antibodies in serum from RVF-infected sheep, it was used as the antigen tested in a validated ELISA on known RVF-positive and RVF-negative sera. Plant-produced mock antigen was also included as a negative control for plant-produced N-protein. Checkerboard titrations of antigen were carried out to optimise the concentration to be used in the ELISA. An optimal N-protein dilution of 1:200, corresponding to 23 μg/ml, was used. For each ELISA, two replicates of positive and negative RVFV control sera and a conjugate control were included. The mean OD values calculated for 3 different batches of the positive control tested ranged from 0.146 to 1.864, while the negative control values ranged from 0.064 to 0.143. The cut-off value to distinguish between positive and negative test outcome in IgG capture ELISA was calculated as the percentage of high-positive control serum (PP) values greater than 7.0. PP values less than 4.0 were regarded as negative while PP values between 4.0 and 7.0 were regarded as suspect. These values were determined from a previous evaluation of a RVFV recombinant N antigen expressed in *E. coli*, used on these same sera and others from different animals [38]. The mean PP values for the positive sera ranged from 53.05 to 186.88, while the negative sera ranged from − 1.52 to 22.27 (Fig. 5).

## Discussion

Rift Valley fever is a re-emerging disease due to the increasing number of mosquito vectors that can spread the RVF virus into non-endemic areas because of recent global warming [39]. Monitoring of the spread of the disease is imperative in both endemic and non-endemic areas, and consequently requires readily available diagnostic tools. The development of a safe and cost-effective diagnostic tool is important, especially for South Africa and other developing countries where RVF is prevalent, and where intensive surveillance for the disease must be conducted [40]. The recent finding from a study in Sudan that infection with Rift Valley fever virus may be associated with miscarriage in pregnant women [41] may increase the urgency and the need for constant surveillance in countries which have both the virus and its mosquito vector.

We investigated the possibility of using *N. benthamiana* to express RVFV N-protein as an antigen for use in an ELISA to distinguish between sera of animals exposed to RVFV and those that were not.

The purified recombinant nucleoprotein expressed in *N. benthamiana* assembled into monomeric (28 kDa), dimeric (56 kDa) and putative pentameric (140 kDa) forms as detected by western blot analysis (Fig. 3). This correlates with work done by Le May et al. [42] who showed similar results with RVFV N-protein expressed in mammalian cells. N-protein expressed in insect cells [43] and in *E. coli* [37] was also shown to assemble into multimeric proteins. It was suggested that the high protein concentration drives the small units of N-protein to assemble into larger stable oligomers [37].

In the current study, use of 197 g of agroinfiltrated leaves resulted in N-protein yields ranging from 500 to 558 μg/g fresh leaf material. These yields are much higher than those previously obtained in transgenic *A. thaliana* (3.8 μg/g of fresh leaf weight) [33], and comparable to those achieved for recombinantly-produced N-protein in *E. coli* of 0.34–0.71 mg/mL [10, 16]. Moreover, sufficient yields of soluble plant-produced N-protein were easily purified for testing.

Mass spectrometry confirmed the presumptive monomeric protein isolated from PAGE-fractionated proteins to be RVFV N-protein. TEM images revealed distinct 10 nm diameter ring-shaped structures (Fig. 4), confirming that the N-protein self-assembled into nucleocapsid-like particles in *N. benthamiana*, which are similar to those observed in *E. coli* (Ferron et al., 2011).

The capacity of the recombinant nucleoprotein (rN) produced in *N. benthamiana* to function as a diagnostic antigen was demonstrated by using it in an ELISA. The rN clearly differentiated between sera of RVFV-infected animals and those from uninfected animals (Fig. 5). The plant-produced N-protein could be used to detect 100% of the known positive sera, with PP values ranging from 53.05 to 186.88. 40% of the known negative sera were detected as negative with PP values ranging from − 1.5 to 3.36, with one suspect sample with a PP value of 4.05, and the remainder (55%) detected as false positive sera with PP values ranging from 8.55 to 22.27 (data not shown). It is possible that the false positives might have arisen as a result of the presence of non-specific proteins present in the plant-produced N-protein preparation, as there were some contaminating plant proteins in the preparation as observed in the Coomassie-stained gel (Fig. 3b). These plant proteins may have reacted non-specifically with antibodies in the sheep serum. However, there were no false negative results measured despite the partial contamination of the antigen with plant proteins.

One of the limitations of the *6 x his* tag is co-purification of the target protein with contaminating proteins which have at least two proximal histidine residues [44]. Contamination of antigen with co-purified proteins was a major obstacle in the purification of human SERCA2a cardiac isoform expressed in *Saccharomyces cerevisiae* [45], and the purification of Gn (glycoprotein N) ectodomain in insect cells [46]. This was resolved by replacing the C-terminal 6xHis-tag with three Strep-tags [23]. High protein purity is required for high specificity and sensitivity in ELISAs [10, 14, 16] and it is preferable to have the plant-produced N-protein more pure so that it can give more accurate ELISA results. However, this work shows preliminary evidence that the plant-produced N-protein can detect RVFV-specific antibodies in sera of sheep. The problem of false positive test results can be addressed partly by optimising the 6xHis-tag protein purification protocol; purifying using Talon resin [45] or replacing the N-terminal 6xHis-tag with strep-tags [23] to encourage reduction of the amount of plant contaminating proteins which are co-purified with the antigen and may react with the plant antibodies in the animal serum.

An advantage of using plant-produced antigens is that they do not require higher biosafety conditions than biosafety level 1 (BSL1) for their production, which is important for diagnostic laboratories that do not have high level biosafety containment facilities. In addition, the method of production in plants is rapid, cheap and scalable [47]. The ELISA technique is ideal for developing countries as it is a low cost, sensitive and rapid technique, and is thus suitable for general surveillance and monitoring animals during RVF outbreaks. Although further evaluation of the plant-produced N protein to show functionality in sera from other sources is required, these results show that it has potential for use as an ELISA reagent to be used to differentiate between infected and vaccinated animals; moreover, it could potentially be used for humans when used with new exploratory vaccines based on RVFV glycoproteins. These advantages could make a significant contribution to a One Health approach towards controlling and managing RVFV infections.

## Methods

### Cloning of Rift Valley fever virus nucleocapsid (N) protein gene into pEAQ-*HT*

The sequence which encodes RVFV nucleocapsid (N-protein) (GenBank accession number JF784 388) was downloaded from GenBank, then human codon optimised, synthesised and cloned into pUC57 to yield a recombinant plasmid pUC57-N by GenScript (GenScript Biotechnologies, Piscataway, NJ, USA). The recombinant plasmid DNA was transformed into *E. coli* DH5α (E. cloni ™, Lucigen, WI) and the transformants were selected for on Luria Bertani (LB) plates containing 100 μg/mL ampicillin. The insert encoding the N-protein was directly cloned from pUC57-N using restriction enzymes *Cfr*91 and *Stu*I into pEAQ-*HT* [48] in-frame with the 6 x histidine tag on the N-terminus of the N-protein sequence to yield pEAQ-*HT*-his-N. The recombinant plasmid was transformed into *E. coli* and the transformants selected on LB plate with 100 μg/mL kanamycin. The recombinant plasmids were confirmed by restriction digest mapping with *Age*I and *Stu*I.

pEAQ-*HT*-his-N was transformed by electroporation into *A. tumefaciens* LBA4404 as described by Maclean et al. [49] and positive colonies selected on LB plates at 27 °C containing kanamycin (50 μg/mL) and rifampicin (50 μg/mL). Positive colonies were confirmed by colony PCR of purified plasmid DNA using pEAQ-specific forward (5’-GACGAACTTGGAGAAAGATTGTTAAGC-3′) and reverse (5’-GACCGCTCACCAAACATAGAAATG-3′) primers. Clones were verified by back-transformation of purified plasmid DNA into *E. coli* cells (Lucigen, US) and selection on LB with kanamycin (50 μg/mL).

### *Agrobacterium* transformation and recombinant protein expression analysis

Small scale transient protein expression was conducted by syringe-infiltrating leaves of 6-week old *N. benthamiana* plants (obtained from BRU seed collection and grown in-house) using recombinant *A. tumefaciens* at an OD_600_ of 0.25, 0.5 and 1 as described by Atkinson et al. [50]. The infiltrated leaves were harvested from 1 to 4 days post infiltration (dpi). The protein was extracted from *N. benthamiana* leaves by grinding up leaves in extraction buffer (100 mM Tris/HCl pH 7.5 and 1% Triton X-100). The crude extracts were clarified by centrifugation at 18407 *g* for 5 min in a bench top centrifuge. The concentration of total soluble protein (TSP) in the crude extract was determined by Bradford assay using BSA (Sigma-Aldrich, MO, USA) as a standard. Equal amounts of protein were loaded onto a 15% SDS-PAGE gel and separated by electrophoresis. The gel was electroblotted onto 0.45 μm nitrocellulose membrane (Amersham ™ Protract) at 15 V for 1 h using a Trans-blot® SD semi-dry blotter (Bio-Rad, California, USA). N-protein was detected with rabbit anti-N antibody (keyhole limpet haemocyanin-linked anti-N peptide (NKPRRMMMKMSEKEG); GenScript, Piscataway, NJ, USA) used at a dilution of 1: 5000 with secondary alkaline phosphatase-conjugated goat anti-rabbit antibody (Sigma, Steinheim, Germany) at 1:7000 dilution. Nitro blue tetrazolium chloro/5-bromo-4 chloro-3-indolyl phosphate (NBT/BCIP) substrate (KPL, Gaithersburg, MD, USA) was used for detection. Protein expression, extraction and western blot analysis were repeated at least three times to confirm the expression of the N-protein.

### Large scale protein expression and purification

Recombinant *Agrobacterium tumefaciens* cultures were grown in LBB medium (tryptone 2.5 g/L, yeast extract 12.5 g/L, NaCl 5 g/L) supplemented with 10 mM MES, pH 5.6, 20 μM acetosyringone, 50 μg/mL kanamycin and 2 mM MgSO_4_ until they reached an OD_600_ of between 3 and 4. *Agrobacterium* cultures were subsequently diluted to an OD_600_ of 0.25 in resuspension medium (10 mM MgCl_2_ and 5 mM MES, pH 5.6). Six-week-old whole *N. benthamiana* plants were placed upside down in the bacterial suspension culture in an airtight steel tank under vacuum of − 90 to − 100 kPa. Following vacuum infiltration, the plants were returned to the plant growth room with 8 h dark, 16 h light at 25 °C with 55–65% humidity until harvesting of leaves at 3 dpi.

#### Ammonium sulphate precipitation

Crude plant protein was extracted from leaves with 100 mM Tris/HCl pH 7.5, 1% Triton X-100 in a 1:2 plant mass: buffer volume ratio using an Ultra-Turrax® homogeniser (IKA® Works Inc., NC, USA) and filtered twice with four layers of Miracloth™ (EMD Millipore Corp., Billirica, MA USA). The crude extract was clarified by centrifugation at 15871×*g*, for 20 min at 4 °C four times or until a pellet was no longer visible. Ammonium sulphate precipitation was performed with a series of increasing (NH_4_)_2_SO_4_ concentrations (0–40%, 40–60% and 60–80%) as described by Englard and Seifter (Englard and Seifter, 1990). The amount of solid (NH_4_)_2_SO_4_ added was calculated using the online tool available at http://www.encorbio.com/protocols/AM-SO4.htm. The (NH_4_)_2_SO_4_ was added to the crude protein slowly and kept at 4 °C, stirring for 1 h. The protein was pelleted by centrifugation at 15871×*g* for 20 min at 4 °C. The 0–40% precipitated pellet was re-suspended with half the original volume of buffer (50 mM Na_2_HPO_4_, 0.5 M NaCl, pH 8) and dialysed overnight at 4 °C using dialysis tubing with a molecular weight cut-off of 10 kDa (Thermo Fischer Scientific, USA) in 2 L of the same buffer. The extract was clarified by centrifugation at 15871×*g* at 4 °C for 20 min. This was repeated four times or until the pellet became invisible.

#### Nickel affinity chromatography

The crude extract was filtered through a 0.45 μm and subsequently a 0.2 μm filter. The protein was loaded on a 5 mL HisTrap column (Sigma-Aldrich, St. Louis, USA) and purified with an automated fast protein liquid chromatography (FPLC) system (ÄKTA Purifier Plus, GE-Healthcare Life Technologies) at a flow rate of 2.5 min/mL. The column was equilibrated with binding buffer (50 mM sodium phosphate, 0.5 M NaCl and 20 mM imidazole, pH 8). N-protein was eluted with 5 column volumes of elution buffer (50 mM sodium phosphate, 0.5 M NaCl, 500 mM imidazole, pH 8). Five mL fractions were collected and absorbance was monitored at 280 nm. Fractions corresponding to the protein peak were analysed for the purity and presence of N-protein by SDS-PAGE with subsequent Coomassie-blue staining (0.1% Brilliant Blue R-250, 50% methanol and 10% glacial acetic acid) and western blotting as described previously. The concentration of the N-protein was quantified by gel densitometry of a Coomassie blue stained gel using a Gene Genius Bioimaging System – GeneTools (version 3.07.03) (Syngene) and BSA (Roche Diagnostics, Germany) was used to construct a linear standard curve.

### Liquid chromatography - mass spectrometry (LC-MS) of N-protein

Following Coomassie blue staining of SDS-PAGE gels, the N-protein monomeric band was excised and sent to the Centre for Proteomic and Genomic Research (Cape Town, South Africa) for LC-MS analysis. The proteins were enzymatically digested with trypsin together with a BSA standard and the resulting peptides were separated by reverse-phase high performance liquid chromatography. The peptides were injected on a Thermo Q-Exactive mass spectrometer (Thermo Fisher Scientific) and the resulting spectra were analysed using Byonic Software (Protein Metrics USA) using reviewed sequences available from UniProt (https://uniprot.org). Gel digested samples were equated against a merged database comprised of *N. benthamiana, N. tabacum, A. tumefaciens* and *Bunyaviridae*.

### Transmission electron microscopy (TEM) of N-protein

The purified protein fractions were applied to copper-coated grids (mesh size 200) which had been made hydrophilic by glow discharging at 25 mA for 30 s using a Model 900 SmartSet Cold Stage Controller (Electron Microscopy Sciences). Three uL of sample was aliquoted onto the grids, which were incubated for 30 s and washed twice with sterile water. The samples were negatively stained for 30 s with 2% (*w*/*v*) uranyl acetate and viewed using a Technai G2 transmission electron microscope (FEI). Particles were measured using the Digital Micrograph software.

### IgG ELISA assays

#### Serum samples

To determine whether the purified RVFV N-protein can be used in an assay to detect RVFV-specific IgG antibodies in sera of animals, the protein was used in ELISA with RVFV-positive and RVFV-negative sera from sheep used for a previous project [25, 38]. The study was approved by the Agricultural Research Council of the Onderstepoort Veterinary Institute (ARC-OVI) in Pretoria, South Africa. A group of 20 seronegative sheep were previously made RVFV-positive by experimental infection with RVFV strain M35/74 at the Agricultural Research Council-Onderstepoort Veterinary Institute (ARC-OVI) [38]. A group of 20 negative reference sera from sheep was included. Origins and other properties of these sera have been described in detail elsewhere [25, 38].

#### Preparation of mock antigen

Mock antigen was prepared by infiltration of *A. tumefaciens* transformed with pEAQ-*HT* plant expression vector lacking any insert into *N. benthamiana.* Crude protein extract was prepared in the same manner as the purified N-protein as described previously, with precipitation using 0–40% (NH_4_)_2_SO_4_ followed by affinity chromatography as described above.

#### Indirect RVFV IgG ELISA

Peak fractions containing the purified N-protein were used in a standard operating procedure (SOP VT_ME_016–03) for RVFV indirect IgG ELISA [25, 38] at the ARC-OVI (Pretoria, South Africa) as a substitute for the recombinant nucleoprotein expressed in bacteria (Ellis et al., 2014; Williams et al., 2011). The test in which the SOP is routinely used is accredited by the South African National Accreditation System (SANAS) and approved by DAFF for use at the ARC-Onderstepoort Veterinary Institute. Briefly, Nunc Polysorp plates (Nunc, Rosklilde, Denmark) were coated with purified recombinant plant-produced N-protein, followed by the addition of different sera and detection of binding by addition of secondary antibody [38]. A total of 40 serum samples were tested (in duplicate) - twenty positive and twenty negative samples [38]. The experiment was conducted three times with three different batches of purified protein.

Optical density (OD) values were read at 405 nm. The net OD_405_ for each serum sample tested was calculated by subtracting the OD_405_ reading of the mock antigen from the cognate reading of that obtained using the plant-produced N antigen to account for any background absorbance in ten separate experiments. The OD readings were converted to PP values (percentage of positive control serum) [38] using the following equation:

$$ \% PP=\frac{Mean\; OD\; of\kern0.17em test\kern0.17em sample- Mean\; OD\; of\kern0.17em negative\kern0.17em control\;}{Mean\; OD\; of\kern0.17em positive\kern0.17em control- Mean\; OD\; of\kern0.17em negative\kern0.17em control}X100 $$

The mean optical densities of the positive and negative controls are calculated based on the duplicate analysis (40 values for each mean) [51].

## Additional file

Additional file 1: **Figure S1.** LC-MS data of N protein: Mass spectrophotometry of N-protein recovered from SDS-PAGE analysis of a nickel affinity chromatography purified protein from infiltrated plant leaf material. Unique peptide sequences are shown in red text. (PPTX 33 kb)

## Abbreviations

BLS1: Biosafety level 1
CPMV: Cowpea mosaic virus
DIVA: Differentiate infected from vaccinated animals
dpi: Days post infiltration
ELISA: Enzyme-linked immunosorbent assay
IgG: Immunoglobulin G
IgM: Immunoglobulin M
LAMP: Loop-mediated amplification
LC-MS: Liquid chromatography mass spectrometry
PP: Percentage positive
TEM: Transmission electron microscopy
VN: Virus neutralisation
