# Supplementary material for: Expression of Rift Valley fever virus N-protein in Nicotiana benthamiana for use as a diagnostic antigen
Source: BMC Biotechnol. 2018 Dec 11;18:77. doi: 10.1186/s12896-018-0489-z (PMC6290525; doi:10.1186/s12896-018-0489-z)
Supplement: Supplementary file 1 — Figure S1. LC-MS data of N protein: Mass spectrophotometry of N-protein recovered from SDS-PAGE analysis of a nickel affinity chromatography purified protein from infiltrated plant leaf material. Unique peptide sequences are shown in red text. (PPTX 33 kb) [file 12896_2018_489_MOESM1_ESM.pptx]

## Slide 1
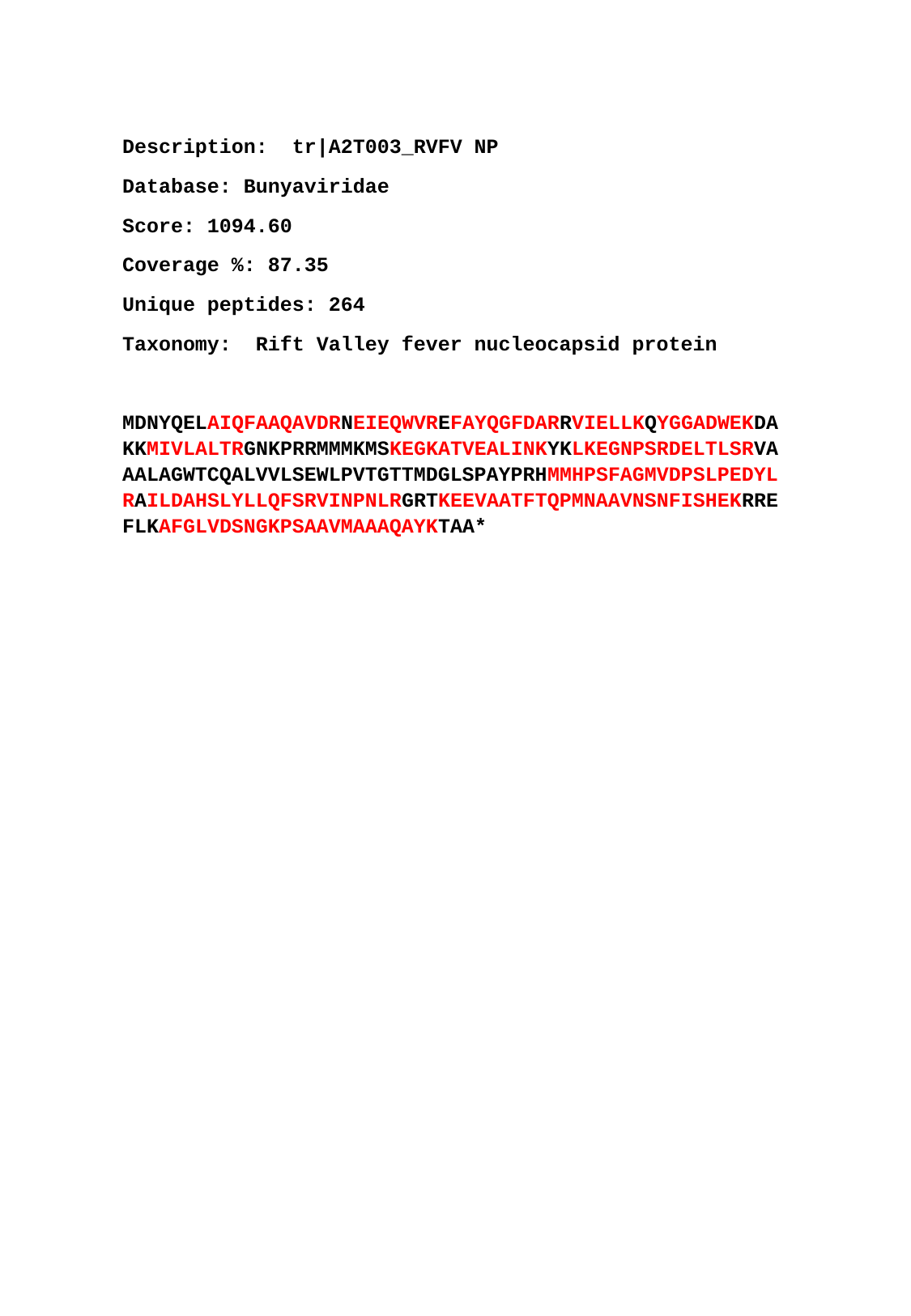

Description: tr|A2T003_RVFV NP
Database: Bunyaviridae
Score: 1094.60
Coverage %: 87.35
Unique peptides: 264
Taxonomy: Rift Valley fever nucleocapsid protein
MDNYQELAIQFAAQAVDRNEIEQWVREFAYQGFDARRVIELLKQYGGADWEKDAKKMIVLALTRGNKPRRMMMKMSKEGKATVEALINKYKLKEGNPSRDELTLSRVAAALAGWTCQALVVLSEWLPVTGTTMDGLSPAYPRHMMHPSFAGMVDPSLPEDYLRAILDAHSLYLLQFSRVINPNLRGRTKEEVAATFTQPMNAAVNSNFISHEKRREFLKAFGLVDSNGKPSAAVMAAAQAYKTAA*
